# Alumni Association

**Published:** 1868-04

**Authors:** S. A. McWilliams

**Affiliations:** Sec’y.


### ALUMNI ASSOCIATION.

MINUTES OF THE MEETING OF THE ALUMNI ASSOCIATION OF CHICAGO MEDICAL COLLEGE, HELD MARCH 3D, 1868.

The members of the Alumni Association of Chicago Medical College met in the lower lecture-room of the College, immediately after the commencement exercises.

The President, Dr. J. S. Jewell, delivered an able address upon medical education, which was listened to with marked attention, and frequently applauded.

On motion, it was the unanimous wish of the alumni that the President be requested to furnish a copy of his address for publication in the CHICAGO MEDICAL EXAMINER.

Some amendments were then made to the Constitution and By-laws.

It was moved, by Dr. Fuller, and carried, that 400 copies of the Constitution and By-laws, as amended, including the President's address, be printed, and a copy sent to each alumnus.

Correspondence, and reading and discussion of papers then followed.

On motion, the Chairman appointed a Committee, consisting of Drs. John M. Woodworth, J. F. Kelsey, and A. W. Gray, to nominate officers, which Committee, after due conference, presented the following names, which were unanimously elected:

*President*—H. P. MERRIMAN, M.D., Chicago.

*1st Vice-President*—N. W. WEBBER, M.D., Naperville, Ill.

*2d Vice-President*—NORMAN BRIDGES, M.D., Chicago.

*Sec'y and Treas.*—S. A. MCWILLIAMS, M.D., Chicago.

The alumni were then notified that the Treasurer was ready to receive their annual dues.

No farther business appearing, the meeting adjourned.

S. A. MCWILLIAMS, M.D., *Sec'y.*

